# Antimicrobial Stewardship Practices in a Subset of Community Pharmacies across the United States

**DOI:** 10.3390/pharmacy11010026

**Published:** 2023-02-02

**Authors:** Yuman Lee, Nicole Bradley

**Affiliations:** Department of Clinical Health Professions, College of Pharmacy and Health Sciences Pharmacy Practice Faculty, St. John’s University, Queens Campus, New York, NY 11439, USA

**Keywords:** antimicrobial stewardship, community pharmacy, community pharmacists

## Abstract

Background: Antimicrobial stewardship in the community is essential as most antibiotic prescribing occurs in the outpatient setting. Pharmacists are recognized as co-leaders for implementing efforts to improve antimicrobial use. Objectives: the purpose of this study is to evaluate current antimicrobial stewardship practices in community pharmacies across the United States and identify perceptions and challenges faced by community pharmacists. Methods: a survey based on the Center of Disease Control and Prevention (CDC) Core Elements of Outpatient Antibiotic Stewardship was created and distributed. Results: Sixty-one community pharmacists participated in the survey. The majority of pharmacists practiced in chain pharmacies. Based on the responses, a minority of pharmacies met the requirements of the CDC core elements: commitment (27.9%), action (24.6%), tracking and reporting (14.8%), and education and expertise (23% for providing pharmacist resources and 9.8% for providing patient resources). Regarding perception, 67.9% felt antimicrobial stewardship is important in the community and would participate in antimicrobial stewardship activities if the opportunity was provided (88.5%). Challenges faced by community pharmacists include the lack of time, staff, training, and technology support; pushback from prescribers and patients; and the lack of leadership, financial incentives, funding, and legal requirements. Conclusions: many challenges exist in community pharmacies inhibiting the full potential of pharmacists in implementing antimicrobial stewardship.

## 1. Introduction

Antibiotic resistance is considered a major public health threat worldwide, with rates of resistance continuing to rise. In the United States (US), more than 2.8 million antibiotic resistant infections occur each year, resulting in over 35,000 deaths [1]. In 2020, there were over 200 million oral antibiotics prescribed in the outpatient setting and it is estimated that at least 28% of outpatient antibiotic prescriptions are completely unnecessary [2,3]. Furthermore, total inappropriate antibiotic use, including unnecessary prescriptions, inappropriate agent, dosing, or duration, is estimated to be approaching 50% of all outpatient antibiotic use [3].

Antimicrobial stewardship is a coordinated set of interventions to improve antibiotic prescribing practices and is considered essential to slowing the spread of bacterial resistance and improving antibiotic related treatment outcomes. The Joint Commission now mandates antimicrobial stewardship programs in all ambulatory care settings in which antibiotics are prescribed [4]. Additionally, the Centers for Disease Control and Prevention (CDC) defines the core elements of outpatient antibiotic stewardship as commitment, action for policy or practice, tracking and reporting, and expertise and education. Community pharmacies and pharmacists are identified as potential partners for outpatient antimicrobial stewardship activities [5]. Despite the CDC’s recommendations and the Joint Commission mandate, no guidance or recommendations for implementing antimicrobial stewardship into community pharmacies exist in the US.

The small, growing body of literature currently exists regarding community pharmacists’perceptions and practices in antimicrobial stewardship. However, all these studies were conducted outside of the US as shown in a systematic review performed by Saha and colleagues [6]. We recognize that the pharmacist’s role and scope of practice may differ outside of the US with previous studies describing the role of community pharmacists outside the US as antimicrobial prescribers for conditions such as urinary tract infections, acute pharyngitis, herpes zoster treatment [7,8,9,10,11], and as leaders in community management of upper respiratory tract infections [12]

The objectives of this study are to evaluate current antimicrobial stewardship practices in community pharmacies across the US and to identify perceptions and challenges that community pharmacists face regarding antimicrobial stewardship.

## 2. Materials and Methods

A 15-item online survey was created using Google’s survey administration software, Google Forms (Supplementary Table S1). Survey questions assessing community pharmacy stewardship practices were designed based on the framework and core elements recommended by the CDC for outpatient antibiotic stewardship. Answer options for survey questions were developed by extrapolating from the examples provided by the CDC guidance for each core element (Supplementary Table S1). Additional questions were included to gather baseline demographics of the participants and identification of perceptions and barriers to antimicrobial stewardship, if any existed. An invitation to participate in the survey was posted to the Facebook group of Pharmacists Moms Group (PhMG^TM^), a professional advocacy organization of women pharmacists in the US, in September 2019. Participation was voluntary and anonymous. Each participant was required to log into their unique Google account to access the survey to prevent duplicate responders. This study received IRB exemption from our institution and permission to post the survey was received from the group administrator. Data were analyzed using descriptive statistics.

## 3. Results

Sixty-one community pharmacists participated in this survey. The majority (85.2%) practiced in chain pharmacy settings and held staff pharmacist positions (54.1%) followed by supervising pharmacist positions (37.7%). Community pharmacists in this survey have been practicing for the following number of years: 1–5 years (26.2%), 6–10 years (26.1%), 11–15 years (23%), 16–20 years (9.8%), and greater than 20 years (4.9%). The majority had no additional postgraduate residency training (95.1%) nor completed an antimicrobial stewardship certification program (98.4%). Most participants were practicing in the northeastern US (27.9%), followed by southeastern US (24.6%), midwestern US (21.3%), southwestern US (19.7%) and western US (6.6%).

**Figure 1:**
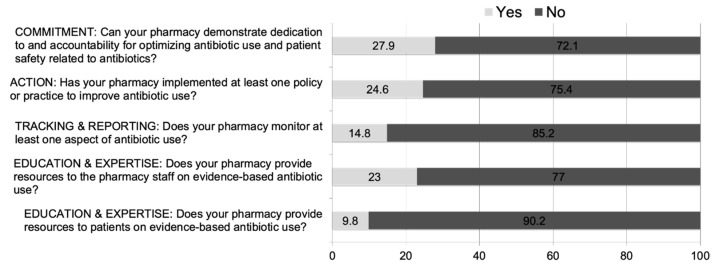
Current Trends of U.S. Community Pharmacies in Meeting CDC’s Core Elements of Outpatient Antimicrobial Stewardship (*n* = 61).

As shown in Figure 1, only a minority of community pharmacies met the CDC Core Elements of Outpatient Antibiotic Stewardship. Approximately 28% reported their pharmacy demonstrated dedication to and accountability for optimizing antibiotic use, 24.6% reported their pharmacy has at least one policy or practice to improve antibiotic use, 14.8% reported monitoring at least one aspect of antibiotic use, 23% reported their pharmacy provides resources to staff on evidence based antibiotic use, and 9.8% provide resources to patients.

When further assessing the antibiotic stewardship practices in the community pharmacies that met the CDC core elements, the following were reported. In the community pharmacies demonstrating dedication to and accountability for optimizing antibiotic use (*n* = 17), 70.6% reported the staff using consistent messages when communicating with the public about the indications for and use of antibiotics, 47.1% reported collaboration with local prescribers and/or other health professionals in the community to improve antibiotic use, 11.8% reported having a single leader assigned to direct antibiotic stewardship activities within their pharmacy, and 5.9% reported that antibiotic stewardship-related duties were included in job descriptions and evaluations. In the community pharmacies that have implemented at least one policy or practice to improve antibiotic use (*n* = 15), 53.3% perform verification of indications and/or durations of antibiotic prescriptions, 26.7% perform follow-up phone calls for patients on antibiotics, 20% encourage prescribers to include diagnosis codes in antibiotic prescriptions, 13.3% perform antibiotic allergy assessments, and 6.7% perform each of the following: point of care testing within the pharmacy, formulary restrictions, and mandatory counseling. In the community pharmacies monitoring at least one aspect of antibiotic use (*n* = 9), the following metrics are tracked: 55.6% the top dispensed antibiotics; 44.4% the durations of antibiotic therapy; 33.3% the antibiotics prescribed based on indications and the number of interventions performed on antibiotics; 22.2% the volume of antibiotic prescriptions dispensed, the top antibiotic prescribers, and the frequency of the same patients on antibiotics; and 11.1% the number of phone calls to prescribers regarding antibiotic issues. In the community pharmacies that provide resources to the pharmacy staff on evidence-based antibiotic use (*n* = 14), 71.4% provide continuing education activities, 57.1% provide timely access to clinical support, and 7.1% provide face-to-face educational training. In the community pharmacies providing resources to patients on evidence-based antibiotic use (*n* = 6), 100% provide patient education on when antibiotics are and are not needed, 83.3% provide patient education on the potential harms of antibiotic use, and 33.3% perform follow-up phone calls for patients on antibiotics.

Despite most pharmacies not meeting the CDC core elements, many respondents felt that antimicrobial stewardship in the community was important or very important (67.2%). Approximately 88.5% reported they would participate in antimicrobial stewardship activities at their pharmacy if provided with the opportunity. Despite the willingness to participate, more than half (55.7%) of respondents reported their pharmacies to have no plans to implement antimicrobial stewardship over the next two years, while 36.1% were unsure of their pharmacy’s plans.

**Figure 2:**
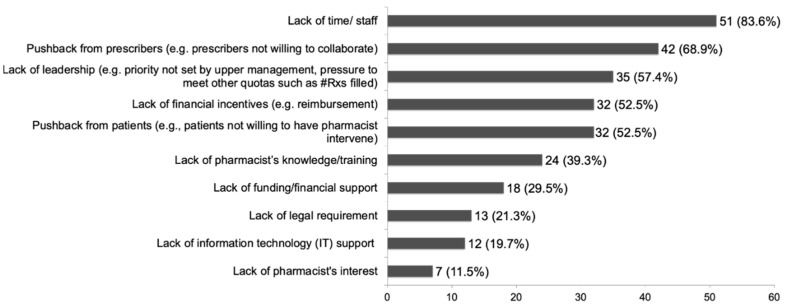
Challenges in Implementing Antimicrobial Stewardship in Community Pharmacies (*n* = 61).

One hundred percent of respondents identified at least one challenge to implementing stewardship in the community setting and 96.7% reported two or more challenges. As seen in Figure 2, common barriers cited were lack of time/staff (83.6%), pushback from prescribers (68.9%), lack of leadership (57.4%), lack of financial incentives (52.5%), and pushback from patients (52.5%). Other barriers included lack of pharmacist training, no legal requirements for participation, and lack of information technology support.

## 4. Discussion

There is a paucity of data investigating the role of community pharmacists in antimicrobial stewardship in the US. Therefore, understanding current trends and barriers to antimicrobial stewardship in the community setting is vital in optimizing the role of community pharmacists as antimicrobial stewards. Our study demonstrates that current practices in community pharmacies are lacking as most do not meet the CDC core elements. Despite this, most community pharmacists feel that antimicrobial stewardship in the community is important and would participate if given the opportunity. We recognize the limitations of our study to be a small sample size, the distribution of our survey among a single group of pharmacists, and the inability to calculate a response rate. Additionally, a validated assessment tool was not used since one does not exist for assessing antimicrobial stewardship practices among community pharmacies. However, we developed our survey questions on the basis of the CDC Core Elements of Outpatient Antibiotic Stewardship in an effort to accurately portray evidence-based practices and best practices for antibiotic stewardship.

Numerous stewardship opportunities for pharmacists exist and may differ based upon the practice’s setting. In the community setting, some stewardship strategies may be more easily implemented than others. For example, the American Society of Health System Pharmacists’ statement on the pharmacist’s role in antimicrobial stewardship suggests providing antibiotic education and counseling to ambulatory care patients and participating in public health initiatives aimed at controlling the spread of infectious diseases as stewardship strategies [13]. Strategies such as these are easily implemented as community pharmacists are already key players in vaccine administration and patient education. In addition to being easily implemented, antibiotic education by community pharmacists has been shown to improve medication adherence [14].

A narrative overview by Bishop and colleagues describes other potential strategies for antimicrobial stewardship in the community setting. They identify five community pharmacist-led stewardship intervention strategies: collaborative practice agreements, point of care testing, patient consultations, academic detailing, and serving as an advocate for patients and other healthcare providers. However, they recognize that implementation of some of these strategies may be challenging and call for additional training in pharmacy schools to help student pharmacists address the expanding role of community pharmacists in outpatient antimicrobial stewardship [15]. In a commentary by Gallagher and colleagues, the importance of integrating stewardship principles into pharmacy education is also emphasized to meet the growing standards of pharmacists as antimicrobial stewards [16].

Collaborative practice agreements (CPAs) allow pharmacists to perform specific patient care functions, such as point of care testing. A cost-effectiveness analysis by Klepser and colleagues demonstrated community pharmacy-based rapid diagnostic testing and treatment for group A Streptococcus pharyngitis provided the most cost-effective and cost-minimizing strategy for the diagnosis and treatment of this common outpatient infectious illness compared to other strategies [17]. However, laws and regulations on collaborative practice agreements vary from state to state. While most states allow CPAs, the scope of these agreements varies extensively, with the broadest legislation allowing pharmacists to provide drug therapy for certain conditions, and others having extremely limited collaborative practice protocols [18]. As a result, the ability of community pharmacists to implement stewardship strategies that rely on CPAs will be dependent on state regulations. Future legislation surrounding CPAs and antimicrobial stewardship needs to recognize the expanding role of community pharmacists in antimicrobial stewardship activities to better utilize community pharmacists in stewardship efforts.

Despite the willingness to participate in and the opportunity for antimicrobial stewardship by community pharmacists, multiple barriers were identified that may impact the ability to implement certain practices. The top three challenges cited by our survey respondents were the lack of time and staff, followed by pushback from prescribers and patients, and the lack of reimbursement for antimicrobial stewardship services. We believe that if antimicrobial stewardship services were billable for reimbursement in community pharmacies, it may inevitably help alleviate issues with time and staffing constraints. Pushback from prescribers and patients may be alleviated through educational campaigns.

## 5. Conclusions

This study reveals the lack of antimicrobial stewardship practices in community pharmacies across the US with the majority of participants not currently meeting the CDC core elements. Community pharmacists are identified as co-leaders of antimicrobial stewardship activities by the CDC and this study demonstrates that the majority of community pharmacists agree that antimicrobial stewardship in the community is important and if given the opportunity, they would participate in stewardship activities. Many challenges exist in the community setting, inhibiting the full potential of pharmacists in stewardship efforts. Some of these barriers include the lack of time and staff, pushback from prescribers, the lack of leadership, the lack of financial incentives, pushback from patients, the lack of training, and the lack of funding. This study highlights the importance and need for addressing these issues as regulations and strategies for antimicrobial stewardship in community settings develop.

## Data Availability

Data supporting reported results can be requested via email to the corresponding author (bradleyn1@stjohns.edu).

## Supplementary Materials

The following supporting information can be downloaded at: https://www.mdpi.com/article/10.3390/pharmacy11010026/s1, Table S1: Survey Tool.
