# Supplementary material for: Antimicrobial Stewardship Practices in a Subset of Community Pharmacies across the United States
Source: Pharmacy (Basel). 2023 Feb 2;11(1):26. doi: 10.3390/pharmacy11010026 (PMC9965000; doi:10.3390/pharmacy11010026)
Supplement: Supplementary file 1 [file pharmacy-11-00026-s001.zip › pharmacy-2177974-supplementary.pdf]

Supplementary Table S1. Survey tool

Pre-survey question

Are you a pharmacist that currently practices in the community setting?

□ Yes □ No

- *If no, survey ends, and participant is prompted with response that they are not qualified to participate in this survey.*

Baseline demographics

1. Which state do you currently practice in? (Drop down menu of 50 states)

2. What community pharmacy setting do you currently practice in?

   □ Chain □ Independent □ Compounding □ Specialty

   □ Retail pharmacy with walk-in medical clinic

3. How long have you been in pharmacy practice?

   □ 1-5 years □ 6-10 years □ 11-15 years □ 16-20 years □ >20 years

4. What is your current role?

   □ Staff pharmacist □ Clinical pharmacist □ Manager/ Supervising pharmacist

   □ Store owner

5. Are you residency trained?

   □ Yes □ No

6. Have you completed an antimicrobial stewardship certification program?

   □ Yes □ No

Antimicrobial Stewardship Practices

Commitment

7. Can your pharmacy demonstrate dedication to and accountability for optimizing antibiotic use and patient safety related to antibiotics?

   □ Yes □ No

   If yes, please indicate which of the following are in place. Select all that apply.

   □ Display of posters, fliers, or other forms of information about antibiotic stewardship

   □ A single leader is assigned to direct antibiotic stewardship activities within your pharmacy

   □ Antibiotic stewardship-related duties are included in job descriptions or evaluations

   □ Staff use consistent messages when communicating with the public about the indications for and use of antibiotics

   □ Collaboration with local prescribers and/or other healthcare professionals in the community to improve antibiotic use

   □ Other, please indicate.

Action

8. Has your pharmacy implemented at least one policy or practice to improve antibiotic use?

   □ Yes □ No

If yes, please indicate which of the following are in place. Select all that apply.

- □ Verification of indications and/or durations of antibiotic prescriptions
- □ Follow-up phone calls for patients on antibiotics
- □ Encourage prescribers to include diagnosis codes in antibiotic prescriptions
- □ Antibiotic allergy assessments (e.g., referral to penicillin-skin testing)
- □ Point of care testing within pharmacy (e.g., rapid influenza testing)
- □ Other, please indicate.

Tracking & Reporting

9. Has your pharmacy implemented at least one policy or practice to improve antibiotic use?
   □ Yes □ No

   If yes, please indicate which of the following are being tracked. Select all that apply.
   - □ Track volume of antibiotic prescriptions dispensed
   - □ Track top dispensed antibiotics (e.g., fast moving antibiotics)
   - □ Track antibiotics prescribed based on indications (e.g., respiratory infections, UTI, etc.)
   - □ Track duration of antibiotic therapy
   - □ Track top antibiotic prescribers
   - □ Track frequency of same patients on antibiotics
   - □ Track number of phone calls made to prescribers regarding antibiotic issues
   - □ Track number of interventions made on antibiotics
   - □ Other, please indicate.

Education & Expertise

10. Does your pharmacy provide resources to the pharmacy staff on evidence-based antibiotic use? □ Yes □ No

    If yes, please indicate how your pharmacy provides antibiotic stewardship education to the pharmacy staff. Select all that apply:
    - □ Provide face-to-face educational training
    - □ Provide continuing education activities
    - □ Provide timely access to clinical support
    - □ Reimbursement of antibiotic stewardship certification program
    - □ Other, please indicate.

11. Does your pharmacy provide resources to patients on evidence-based antibiotic use?
    □ Yes □ No

    If yes, please indicate how your pharmacy provides antibiotic stewardship education to patients. Select all that apply.
    - □ Provide patient education on when antibiotics are and are not needed
    - □ Provide patient education on the potential harms of antibiotic use
    - □ Follow-up phone calls for patients on antibiotics
    - □ Other, please indicate.

Perceptions on Antimicrobial Stewardship in the Community

12. How important is antibiotic stewardship in the community?
    □ Not important at all □ Low importance □ No opinion □ Important □ Very important

13. If provided the opportunity, how likely are you to participate in antimicrobial stewardship activities in your pharmacy?
    □ Very unlikely □ Unlikely □ Undecided □ Likely □ Very likely

14. Does your pharmacy have anticipated plans for the implementation of antibiotic stewardship within the next 2 years?
    □ Yes □ No □ I'm not sure

15. What challenge(s) do you face or foresee in implementing antimicrobial stewardship in community pharmacy? Select all that apply.
    □ Lack of pharmacist's interest
    □ Lack of time/staff
    □ Lack of pharmacist's knowledge/training
    □ Lack of financial incentives (e.g., reimbursement)
    □ Lack of funding/financial support
    □ Lack of legal requirement
    □ Lack of information technology (IT) support
    □ Lack of leadership (e.g., priority not set by upper management, pressure to meet other quotas such as #Rxs filled)
    □ Pushback from patients (e.g., patients not willing to have pharmacist intervene)
    □ Pushback from prescribers (e.g., prescribers not willing to collaborate)
    □ Other, please indicate.
